# Modelling and Control of a Reconfigurable Robot for Achieving Reconfiguration and Locomotion with Different Shapes

**DOI:** 10.3390/s21165362

**Published:** 2021-08-09

**Authors:** S. M. Bhagya P. Samarakoon, M. A. Viraj J. Muthugala, Raihan E. Abdulkader, Soh Wei Si, Thein T. Tun, Mohan Rajesh Elara

**Affiliations:** Engineering Product Development Pillar, Singapore University of Technology and Design, 8 Somapah Rd, Singapore 487372, Singapore; bhagya_samarakoon@mymail.sutd.edu.sg (S.M.B.P.S.); raihan619@gmail.com (R.E.A.); weisi_soh@mymail.sutd.edu.sg (S.W.S.); thantun_thein@mymail.sutd.edu.sg (T.T.T.); rajeshelara@sutd.edu.sg (M.R.E.)

**Keywords:** robot controlling, reconfigurable robotics, kinematics, area coverage

## Abstract

Area coverage is a crucial factor for a robot intended for applications such as floor cleaning, disinfection, and inspection. Robots with fixed shapes could not realize an adequate level of area coverage performance. Reconfigurable robots have been introduced to overcome the limitations of fixed-shape robots, such as accessing narrow spaces and cover obstacles. Although state-of-the-art reconfigurable robots used for coverage applications are capable of shape-changing for improving the area coverage, the reconfiguration is limited to a few predefined shapes. It has been proven that the ability of reconfiguration beyond a few shapes can significantly improve the area coverage performance of a reconfigurable robot. In this regard, this paper proposes a novel robot model and a low-level controller that can facilitate the reconfiguration beyond a small set of predefined shapes and locomotion per instructions while firmly maintaining the shape. A prototype of a robot that facilitates the aim mentioned above has been designed and developed. The proposed robot model and controller have been integrated into the prototype, and experiments have been conducted considering various reconfiguration and locomotion scenarios. Experimental results confirm the validity of the proposed model and controller during reconfiguration and locomotion of the robot. Moreover, the applicability of the proposed model and controller for achieving high-level autonomous capabilities has been proven.

## 1. Introduction

Large buildings are extensively constructed throughout the world due to urbanization [1]. Maintenance and cleaning of these buildings should be conducted routinely. The growth of buildings increases the areas to be cleaned. Furthermore, the socioeconomic background and the busy day-to-day lifestyles of people make these maintenance tasks complicated. The scarcity of laborers is another major concern faced by developed countries. Cleaning robots can be introduced as a solution to these issues. According to the literature, many robots have been designed and developed for cleaning applications, such as staircase cleaning [2], floor cleaning [3], wall cleaning [4], pool cleaning [5], furniture [6], and duct cleaning [7]. Among these robots, floor cleaning robots have a higher demand since floors are required to be cleaned more often than other entities.

Area coverage is a crucial factor for a floor cleaning robot [8,9]. Many attempts to improve area coverage of floor cleaning robots can be found in the literature. In this regard, an evolutionary-based navigation method for improving area coverage of a floor cleaning robot has been proposed in [10]. The coverage method is executed by dividing the environment into circular grid cells where a cell’s size is equivalent to the robot’s size. Miao et al. [11] proposed a map segmentation and spiral-based coverage method to reduce the computational complexity of planning for large environments. A method to solve deadlock problems during complete area coverage in a dynamic environment has been proposed [12]. The work [13] introduced a scheduling method for a cleaning robot with multiple cleaning cycles to improve productivity. The proposed method simulates the dust distribution and the path planning of the robot to find an optimum schedule for activating different cleaning cycles. A bio-inspired neural network model to improve the area coverage efficiency using multiple robots has been examined in [14]. The time taken for the coverage is lesser in the case of multiple robots compared to a single robot. On the other hand, the cost of deployment is high since multiple robots are required.

The coverage methods discussed above have been proposed for robots with fixed shapes. The main shortcomings of fixed-shape robots are the incapability of accessing narrow spaces and inefficiency of coverage. Reconfigurable robots have been introduced to cope with the shortcomings of fixed-shape robots in a wide range of application domains, such as exploration and inspection [15,16,17]. The survey [17] revealed many existing developments and future challenges of modular self-reconfigurable robots. According to the survey, less work has been conducted on developing modular-reconfigurable robots for area coverage applications. The reconfigurable robot could be classified into two major classes; intra-reconfigurable: a single entity robot that can change the morphology and inter-reconfigurable: individual robots/modules that can assemble to form a new configuration [18]. The majority of the reconfigurable robots introduced for floor cleaning applications are intra-reconfigurable [18,19,20,21]. The coverage performance improvement of these reconfigurable robots over fixed-shape robots has been evaluated in [19]. The study proves that reconfigurable robots can outperform fixed-shape robots. Extensive developments of various aspects of these reconfigurable robots, such as path planning methods, tradeoff of performance factors, and hardware improvements, can be found [22,23,24,25].

**Figure 1:**
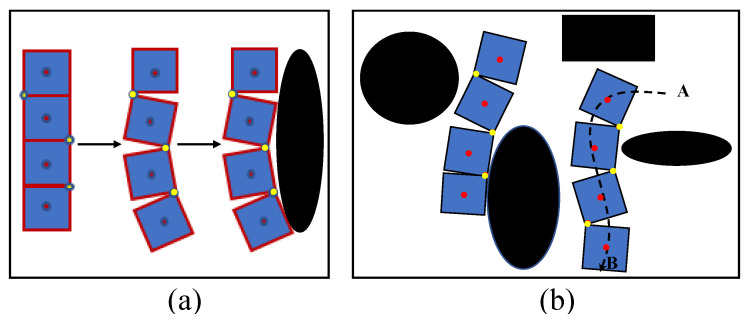
(**a**) A reconfigurable robot changes its shape according to the obstacle and navigates toward the obstacle for coverage. (**b**) Robot changes its shape to navigate through obstacles.

Although the above-mentioned robots are reconfigurable, the reconfiguration is limited to a small set of predefined shapes (e.g., hTetrakis: three shapes [21], hTetro: seven shapes [19,25,26], hHoneycomb: seven shapes [20], and hTrihex: two shapes [27]). According to [28,29,30], area coverage performance of a reconfigurable robot can be improved by considering beyond a small set of predefined shapes for the reconfiguration. The main argument of the work can be explained with the aid of the examples shown in Figure 1a,b, which represent two scenarios where a robot is covering obstacles to maximize the coverage and the robot navigates through obstacles while changing its shape. In contrast, if the same robot had considered only a small set of predefined shapes (i.e., similar to 7 shapes for hTetro [19,25,26]) for the reconfiguration, the robot would have failed in achieving the same level of performance. Handling this sort of reconfiguration and locomotion of a reconfigurable robot through low-level modeling and control is challenging. However, the work in [28,29,30] is limited to high-level simulations that assess the coverage performance, and the low-level modeling and controlling required for achieving the desired goals have not been discussed in the scope.

The majority of floor cleaning reconfigurable robots such as hTetrakis [21], hHoneycomb [20], hTrihex [27], and initial versions of hTetro [19] have active hinges for reconfiguration. The reconfiguration of the robots with active hinges is carried out through the operation of the servo motors attached to the hinges, and no kinematic for drive mechanisms is utilized for the reconfiguration. Nevertheless, the servo motors attached to active hinges are often prone to failures in the long run since the hinge actuators have to bear excessive loads during reconfiguration as well as torque acting on them from other blocks during the navigation. Even though the robots with active hinge mechanisms facilitate the ease of reconfiguration to any shape, the existing kinematic models and controllers of those robots are not designed to facilitate the navigation with any shape other than a set of predefined discrete shapes. The work in [25,26] proposed two variants of hTetro with passive hinges; hTetro with differential drive locomotion and steering locomotion mechanism, respectively. The robot models presented in the cited work are capable of reconfiguring a robot through the motion of drive mechanisms. However, in the robot models considered, the robot locomotes only with the predefined seven shapes. Apart from that, no attention has been paid to firmly maintain the robot’s shape configuration by controlling actions during the locomotion. The robot’s shape configuration is locked after reconfiguration into one of the discrete shapes using the electromagnet-based locking mechanisms attached to the sides of the robot’s blocks. The requirement of electromagnet-based locking mechanisms clearly exposes the limitation of the controllers proposed in [25,26] in maintaining a shape configuration during the locomotion. Furthermore, some of these robots’ locomotion hardware designs have limited movement ability with any shape configuration. Particularly, the robot models of those robots would often encounter singularities due to the design when the robot attempted to locomote while maintaining any shape configuration other than the set of predefined discrete shapes.

Based on the facts discussed above, it is evident that the existing kinematic models and controllers of reconfigurable robots consider that the robots are reconfigured only into a small number of predefined shapes, and the existing models cannot be used to reconfigure and locomote a robot if it reconfigures to any shape beyond a set of predefined shapes. Furthermore, none of the existing work has paid attention to developing a robot model and controller to maintain a robot’s configuration during navigation since the rigidity is provided by locking mechanisms.

Therefore, this paper proposes a novel robot model and a controller for a reconfigurable robot to reconfigure into any possible shape and locomote with the shape configuration. The facilitation of the locomotion with any possible shape configuration without limiting to a small set of predefined shapes is the major contribution of the proposed model to the state-of-the-art. The proposed robot model and controller are presented in Section 2. Experimental results are analyzed and discussed in Section 3. Section 4 concludes the work.

## 2. Robot Model and Controlling

### 2.1. Hardware Platform

**Figure 2:**
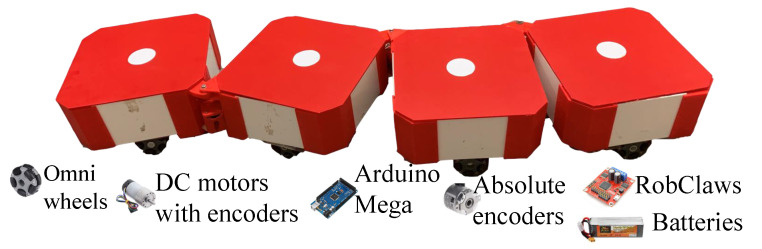
Hardware platform.

The platform of the robot consists of four blocks, which are serially connected through passive hinges. A prototype of the robot and its main hardware components are shown in Figure 2. These three hinges enable the relative motion between blocks that support the reconfiguration into various shapes. Each hinge is connected with an absolute encoder with 1${}^{\circ }$ resolution to monitor the hinge status. These encoders are equipped with an asynchronous serial (TTL) interface that returns the exact position of a hinge. The range of movement of hinges is considered as 0${}^{\circ }$ to 180${}^{\circ }$. The dimensions of each block are 25 cm × 25 cm × 13 cm (length × width × height). Each block consists of four-wheel omni drive powered by 6 V DC motors with omnidirectional wheels (diameter of 56 mm). The drive motors have a nominal speed of 56 rpm and consist of incremental encoders (8256 counts per revolution). An Arduino mega controller is used to implement the low-level controlling functionalities of the robot. The hinge encoders are communicated with the microcontroller using an asynchronous serial communication protocol. The data transfer rate of this communication link is set to 38,400 bits per second (baud rate). This transfer rate is fast enough to properly capture the hinge rotations. Drive motors are controlled through eight motor controllers (Roboclaw 2 × 7.5 A) connected to the microcontroller through a serial connection. The encoders of the drive motors are connected to the motor controllers, and quadrature decodings are performed within the motor controllers. The robot is powered by 11.1 V LiPo batteries.

### 2.2. Locomotion Kinematic Model (LKM)

**Figure 3:**
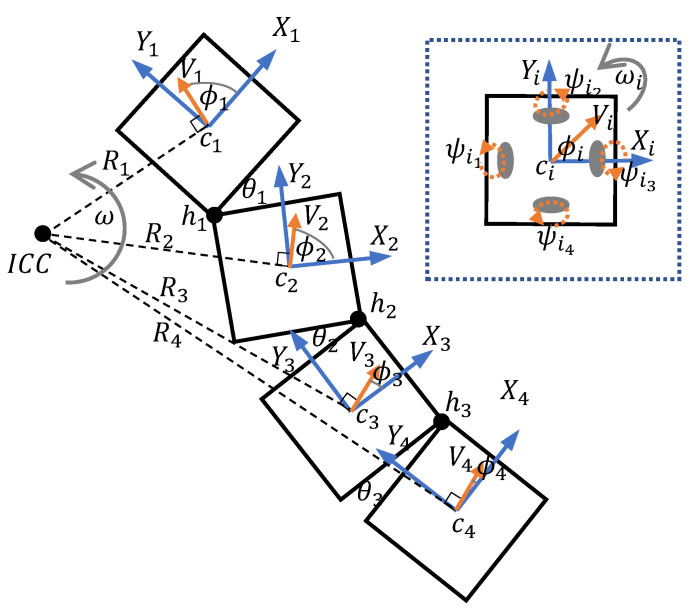
Locomotion kinematic model of the robot.

A scenario of the robot with a shape configuration, where the ith hinge (i.e., $h_{i}$) of the robot has an angle of $\theta_{i}$ for i=1,2,3 is depicted in Figure 3. $c_{i}$ represents the center of the ith block, and the ith frame $\left\{X_{i},Y_{i}\right\}$ is associated with the ith block for i=1,2,3,4. Block 1 of the robot is considered the reference, and it is assumed that all the high-level autonomy functionalities of the robot are performed with respect to the 1st frame.

The magnitude of the linear velocity (i.e., $V_{i}$), the direction of the linear velocity (i.e., $\phi_{i}$), and the angular velocity (i.e., $\omega_{i}$) of the ith block could individually be controllable since the ith block consists of an omni-drive mechanism. However, these parameters should be coordinated for proper locomotion of the robot since the blocks are mechanically connected through passive hinges. Otherwise, the morphology would not be possible to maintain in rigid form. It is expected that the autonomy layer issues $V_{1}$, $\phi_{1}$, and $\omega_{1}$ for accomplishing high-level functionalities such as locomotion for area coverage since block 1 is the reference block. Thus, $V_{i}$, $\phi_{i}$, and $\omega_{i}$ for i=2,3,4 should be determined for the proper locomotion of the robot while maintaining the morphology firm.

In the case of $\omega_{1}\neq 0$, the whole structure of the robot should be moved around a single point known as the Instantaneous Center of Curvature (i.e., ICC) with the angular velocity of $\omega =\omega_{i}$∀i, to maintain the morphology firm. The direction of $R_{1}$, which runs from the center of block 1, $c_{1}$ to ICC, should be perpendicular to $V_{1}$. Thus, the magnitude and direction of $R_{1}$ can be determined as in (1). Then, the coordinates of ICC with respect to the 1st frame, ${}^{1}ICC$ can be determined as in (2). The magnitude and direction of the vector connecting the center of block *i* to ICC, $R_{i}$ can be determined as in (3) for i=2,3,4 since the position of ICC is found in the previous step. The center of block *i* with respect to the 1st frame, ${}^{1}c_{i}$ can be found from (4), where *T* is the homogeneous transformation matrix between the ith and the (i+1)th frame given in (5). $t_{X_{i}}$ and $t_{X_{i}}$ are the translations of (i+1)th frame along $X_{i}$ and $Y_{i}$ axes.

(1) $$\begin{array}{c} |R_{1}|\;=\frac{|V_{1}|}{|\omega_{1}|}\\ \;\;\;\;\;\;\;\;\;\;\;\;\;∠R_{1}=\phi_{1}+\frac{\pi }{2}\mathrm{sign}\left(\omega_{1}\right) \end{array}$$

(2) $${}^{1}ICC={\left[\begin{array}{cc} |R_{1}|\cos\left(∠R_{1}\right) & |R_{1}|\sin\left(∠R_{1}\right) \end{array}\right]}^{T}$$

(3) $$\begin{array}{c} |R_{i}\left|\;=\;\right|\vec{{}^{1}c_{i}{}^{1}ICC}|\;\;\;\;\;\;\;\;\;\;\;\;\mathrm{for}\;i=2,3,4\\ ∠R_{i}=\mathrm{atan}2\left({}^{1}ICC_{Y}-{}^{1}c_{i_{Y}},{}^{1}ICC_{X}-{}^{1}c_{i_{X}}\right)\;\;\;\;\;\;\;\;\;\;\;\;\;\; \end{array}$$

(4) $$\left[\begin{array}{c} {}^{1}c_{i}\\ 1 \end{array}\right]=\prod_{k=1}^{i-1}\;\;{}_{k+1}^{k}T\;\;{\left[0\;0\;1\right]}^{T}\;\;\;\;\mathrm{for}\;i\;=\;2,\;3,\;4$$

(5) $${}_{i+1}^{i}T=\left[\begin{array}{ccc} \cos\theta_{i} & -\sin\theta_{i} & t_{X_{i}}\\ \sin\theta_{i} & \cos\theta_{i} & t_{Y_{i}}\\ 0 & 0 & 1 \end{array}\right]$$

The magnitude and direction of the linear velocity of block *i* (i.e., $V_{i}$ and $\phi_{i}$, respectively) can be determined from (6). By using (1) to (6), all the controllable parameters of each block consisted of an omni-drive mechanism can be found. The angular velocities of the motors attached to the drive wheels of a block should be commanded to vary accordingly to configure the block to have the desired parameters. The Block Kinematic Model (BKM) given in (7) for block *i* is used for determining the reference angular velocity of the kth wheel (i.e., $\psi_{i_{k}},\;k=1,2,3,4$) of block *i* (refer the wheel placements given in Figure 3). Here, *L* is the distance between the wheel center to the center of the block, and *r* is the radius of a wheel.

(6) $$\begin{array}{c} |V_{i}\left|\;=\;\right|R_{i}\left|\right|\omega_{i}|\;\;\;\;\;\;\;\;\;\mathrm{for}\;i=2,3,4\\ \phi_{i}=∠R_{i}-\frac{\pi }{2}\mathrm{sign}\left(\omega \right)\;\;\;\;\;\;\;\;\;\;\;\;\;\;\; \end{array}$$

(7) $$\left[\begin{array}{c} \psi_{i_{1}}\\ \psi_{i_{2}}\\ \psi_{i_{3}}\\ \psi_{i_{4}} \end{array}\right]=\frac{1}{r}\left[\begin{array}{ccc} 0 & 1 & -L\\ 1 & 0 & -L\\ 0 & -1 & -L\\ -1 & 0 & -L \end{array}\right]\left[\begin{array}{c} |V_{i}|\cos\phi_{i}\\ |V_{i}|\sin\phi_{i}\\ \omega_{i} \end{array}\right]$$

If $\omega_{1}=0$, then the robot does not have a rotation. Thus, the derivations of reference $V_{i}$, $\phi_{i}$, and $\omega_{i}$ could not be performed using (1) to (6). In this case, the linear velocities of each block should be the same as the 1st block in terms of magnitude and direction with respect to the inertial frame. These linear velocity components of the block *i* can be found from (8) for i=2,3,4. The corresponding reference angular wheel velocities of block *i* can be obtained from (7).

(8) $$\left[\begin{array}{c} V_{i}\cos\phi_{i}\\ V_{i}\sin\phi_{i}\\ 0 \end{array}\right]=\prod_{k=1}^{i-1}\;\;{}_{k+1}^{k}T^{-1}\left[\begin{array}{c} V_{1}\cos\phi_{1}\\ V_{1}\sin\phi_{1}\\ 0 \end{array}\right]\;\mathrm{for}\;i\;=\;2,\;3,\;4$$

### 2.3. Reconfiguration Kinematic Model (RKM)

**Figure 4:**
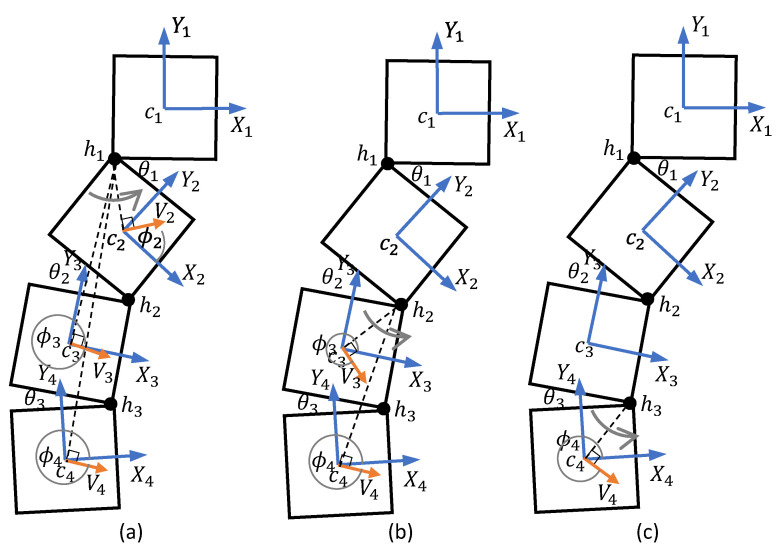
Kinematic models for reconfiguration. (**a**) For varying hinge 1, (**b**) for varying hinge 2, and (**c**) for varying hinge 3.

The four blocks of the robot are interconnected through passive hinges. Thus, the reconfiguration should only be achieved by the relative movements of the blocks through the drive mechanisms. For the sake of reducing complexity, it is assumed that the positioning of only one hinge is performed at a time. Reconfigurations through hinge 1, 2, and 3 during an arbitrary configuration of the robot are depicted in Figure 4a–c, respectively. Furthermore, the reconfiguration strategy has been formulated in such a way that the reconfiguration does not alter the position and orientation of block 1 since block 1 is the reference.

In the case of reconfiguration through $h_{1}$, the block 2, 3, and 4 should rotate around $h_{1}$. For proper coordination, angular velocities of all the moving blocks should be the same ($\omega_{i}=-\dot{\theta_{1}}$, for i=2,3,4). To make the ICC of each moving block on $h_{1}$, the direction of the linear velocity of block *i*, $\phi_{i}$ should be perpendicular to $\vec{c_{i}h_{1}}$, and the radius of rotation should be $|\vec{h_{1}c_{i}}|$. Thus, the magnitude and the direction of the linear velocity of the ith block for reconfiguration around $h_{1}$ can be obtained from (9) for i=2,3,4. The linear and angular velocity of block 1 (i.e., $V_{1}$ and $\omega_{1}$) should be kept at zero.

(9) $$\begin{array}{c} |V_{i}\left|\;=\;\right|\vec{h_{1}c_{i}}\left|\right|\omega_{i}|\;\;\;\;\;\;\;\;\;\;\mathrm{for}\;i=2,3,4\\ \phi_{i}=∠\vec{c_{i}h_{1}}-\frac{\pi }{2}\mathrm{sign}\left(\omega_{i}\right)\;\;\;\;\;\;\;\;\;\;\;\;\;\;\; \end{array}$$

The hinge angle, $\theta_{2}$, can be varied by moving blocks 3 and 4 while keeping block 1 and 2 at a standstill. The ICC of blocks 3 and 4 should be on $h_{2}$ with the same angular velocity ($\omega_{i}=\dot{\theta_{2}}$, for i=3,4) in this regard. As similar to the previous case, the direction of the linear velocity of block *i*, $\phi_{i}$ should be perpendicular to $\vec{C_{i}h_{2}}$, and the radius of rotation should be $|\vec{h_{2}c_{i}}|$, for i=3,4. The magnitude and the direction of the linear velocity of the ith block for reconfiguration around $h_{2}$ can be obtained from (10) for i=3,4. The linear and angular velocities of blocks 1 and 2 should be kept zero.

(10) $$\begin{array}{c} |V_{i}\left|\;=\;\right|\vec{h_{2}c_{i}}\left|\right|\omega_{i}|\;\;\;\;\;\;\;\;\;\;\mathrm{for}\;i=3,4\\ \phi_{i}=∠\vec{c_{i}h_{2}}-\frac{\pi }{2}\mathrm{sign}\left(\omega_{i}\right)\;\;\;\;\;\;\;\;\;\;\;\; \end{array}$$

For the reconfiguration through $h_{3}$, the magnitude and direction of the linear velocity of block 4 can be configured as in (11), similar to the reconfiguration around $h_{1}$ and $h_{2}$. The linear and angular velocities of the other three blocks should be kept at zero.

(11) $$\begin{array}{c} |V_{i}\left|\;=\;\right|\vec{h_{3}c_{i}}\left|\right|\omega_{i}|\;\;\;\;\;\;\;\;\;\;\mathrm{for}\;i=4\\ \phi_{i}=∠\vec{c_{i}h_{3}}-\frac{\pi }{2}\mathrm{sign}\left(\omega_{i}\right)\;\;\;\;\;\;\;\;\;\; \end{array}$$

### 2.4. Control Architecture

**Figure 5:**
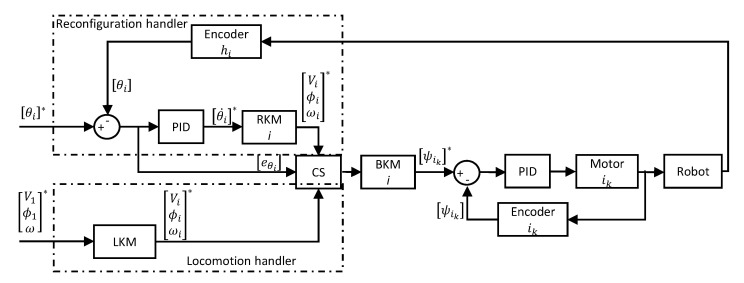
Control architecture.

The control architecture of the robot is depicted in Figure 5 as a block diagram. It is expected that the morphology to be reconfigured is provided to the system as the set of reference hinge angles (i.e., ${\left[\theta_{i}\right]}^{*}$, for i=1,2,3) by the high-level autonomy layer. It is also considered that the reference magnitude and direction of linear velocity and angular velocity of block 1 (i.e., $V_{1}^{*}$, $\phi_{1}^{*}$, and $\omega_{1}^{*}$) are provided by the high-level control layer to locomote the robot as per the requirement. Thus, the goals of the controller are to reconfigure the robot to a given morphology and move the robot while keeping the morphology firm. The system consists of two sub handlers; a reconfiguration handler and locomotion handler, to achieve these goals.

In the reconfiguration handler, the error between a reference hinge angle (i.e., ${\left[\theta_{i}\right]}^{*}$) and current hinge position (i.e., $\left[\theta_{i}\right]$) measured from the corresponding hinge encoder (i.e., $h_{i}$) is calculated as $\left[e_{\theta_{i}}\right]$. A PID controller determines a reference angular velocity for each hinge joint (i.e., ${\dot{\theta_{i}}}^{*}$) to configure it to the reference angle. It should be noted that there are three PID controllers for the three hinges. Then, RKM determines the reference linear and angular velocities of each block (i.e., ${\left[V_{i}^{*}\;\phi_{i}^{*}\;\omega_{i}^{*}\right]}^{T}$) of the robot corresponding to the incoming ${\left[\dot{\theta_{i}}\right]}^{*}$. Initially, the references from the reconfiguration handler are passed to the BKM, which determines the reference angular velocities of wheels of each block ${\left[w_{i_{k}}\right]}^{*}$. A PID controller is used with each drive wheel to achieve the desired velocity control of the motors attached to the corresponding wheels. The encoders fixed to the drive motors are used to take the feedback of the angular velocities of wheels. The operation of the reconfiguration handler is ceased after settling $\left[e_{\theta_{i}}\right]\approx \left[0\right]$ for an experimentally decided threshold time ($t_{T}$). After this instance, the system gives priority to the locomotion handler. This shifting of control is performed by the Controller Selector (CS).

After the reconfiguration of the robot, the locomotion of the robot per the incoming instruction (i.e., ${\left[V_{1}^{*}\;\phi_{1}^{*}\;\omega_{1}^{*}\right]}^{T}$ is performed. The LKM determines the corresponding reference control parameters for each block of the robot (i.e., ${\left[V_{i}^{*}\;\phi_{i}^{*}\;\omega_{i}^{*}\right]}^{T}$). Similar to the reconfiguration stage, these parameters are fed to the BKM to drive the robot. The locomotion of the robot might cause adverse effects on maintaining the firmness of the morphology due to external disturbances and impreciseness of the controlling, such as errors in wheel velocities. Moreover, the hinge angles might be altered during the locomotion. Thus, the error between the reference hinge angle and the current hinge positioning of the ith hinge, $e_{\theta_{i}}$ is continuously monitored throughout the locomotion. If this error exceeds an experimentally decided threshold, the priority is given back to the reconfiguration handler by ceasing the locomotion. Then, the reconfiguration handler corrects the error by again reconfiguring. This shifting is performed by the CS by observing $e_{\theta_{i}}$.

## 3. Results and Discussion

### 3.1. Experimental Setup

Two sets of experiments have been conducted to verify the proposed robot model and controller. In the first set of experiments, the reconfiguration ability of the robot has been analyzed. The locomotion ability of the robot with different shape configurations has been analyzed in the second set of experiments. An overhead camera was used to record the movements of the robot during the test cases. The videos were recorded in 720P resolution with 60 fps. Trackable markers were placed on the robot, and the recorded videos were examined using Kinovea (www.kinovea.org, accessed on 24 April 2021) software for motion analysis. The cycle time of the main controlling loop of the robot is 66 ms. An explanatory video compiled based on the experiments can be found in the Supplementary multimedia attachment.

### 3.2. Validation of Reconfiguration

**Figure 6:**
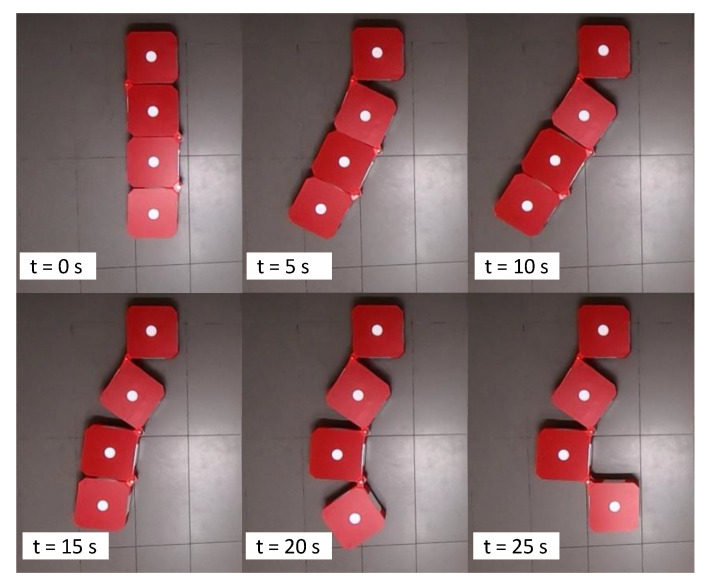
Snapshots taken during a reconfiguration of the robot.

**Figure 7:**
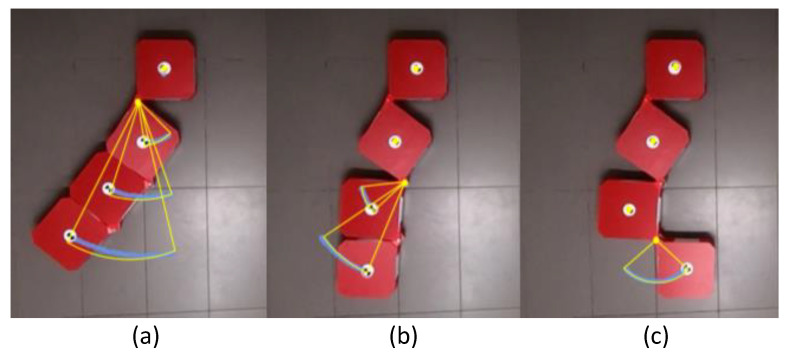
Motion analysis of the reconfiguration. The traced paths are annotated in blue. The expected movement paths are given in yellow. Reconfigurations through hinge 1, 2, and 3 are given in (**a**–**c**), respectively.

Snapshots taken during a reconfiguration test case are given in Figure 6. Here, the robot was commanded to reconfigure into ${\left[35^{\circ }\;20^{\circ }\;90^{\circ }\right]}^{*T}$. After the ceasing of the reconfiguration handler, the actual hinge angles were ${\left[37^{\circ }\;23^{\circ }\;90^{\circ }\right]}^{T}$, which indicate a 2${}^{\circ }$ and 3${}^{\circ }$ error of reconfiguration in hinge 1 and 2, respectively. The Root Mean Square Error (RMSE) error was 2.08${}^{\circ }$ in this case. The results of movement tracking are given in Figure 7. During the reconfiguration of hinge 1, it can be observed that the center of hinge 1 remained motionless as expected. However, a slight deviation (2 cm) of $c_{1}$ along the robot’s Y-axis could be observed due to the forces acting on block 1 during the reconfiguration. This deviation can be corrected by a high-level autonomy layer that localizes the robot on the world frame. The centers of blocks 2, 3, and 4 were moved in circular paths centering $h_{1}$, which is the requirement for the proper reconfiguration from $h_{1}$. In the events of hinge 2 and 3, the traced movements were compliant with the expectations as similar to the event of hinge 1 (see Figure 7b,c).

Similarly, 10 heterogeneous reconfiguration test cases were analyzed, and the robot was capable of successfully performing the reconfiguration as instructed. The overall RMSE was 2.17${}^{\circ }$, suggesting a lower error in reconfiguration. Therefore, it can be concluded that the first set of experiments confirms the ability of the proposed robot model and the controller in reconfiguring the robot to a given configuration with an adequate level of performance.

### 3.3. Validation of Locomotion

**Figure 8:**
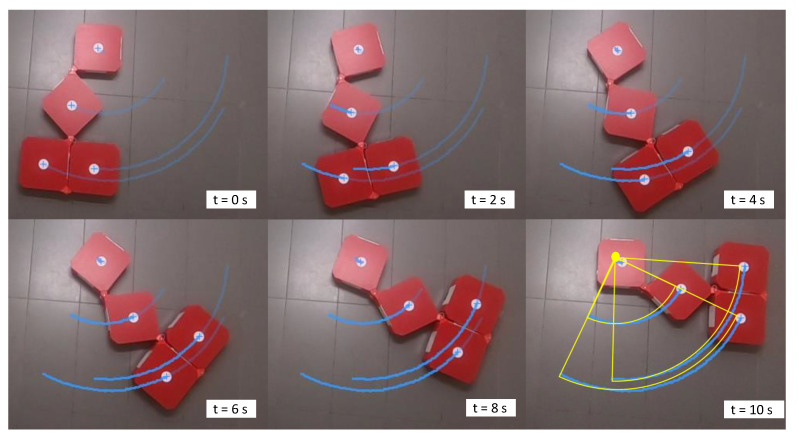
Snapshots taken and the motion analysis during the locomotion with $V_{1}=0$, $\phi_{1}=0$, and $\omega_{1}=0.154$ rad/s. The traced paths are annotated in blue. The expected movement paths are given in yellow.

**Figure 9:**
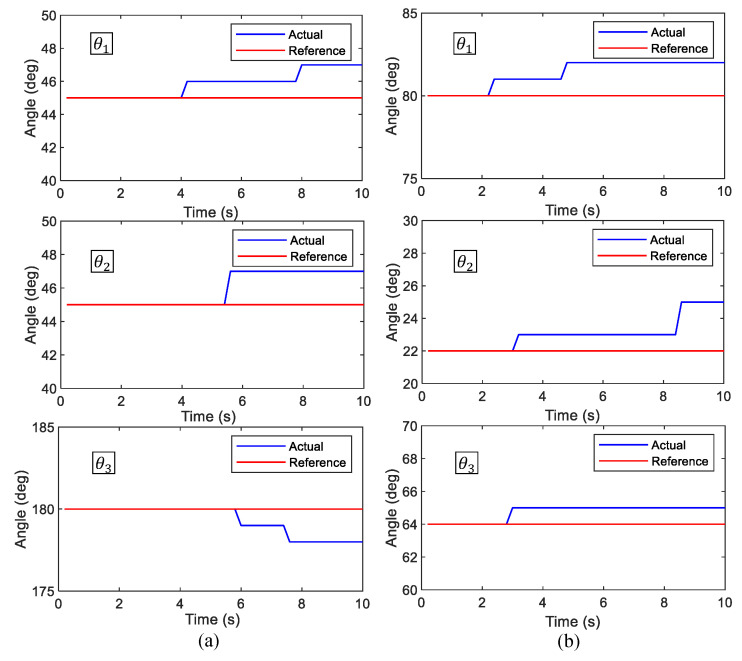
The variation of the hinge angles measured through hinge encoders during the sample locomotion cases. (**a**): case 1, and (**b**): case 2.

Locomotion of the robot while maintaining a given shape configuration of the robot has been evaluated in this set of test cases. In the first sample case, the robot had been reconfigured to ${\left[45^{\circ }\;45^{\circ }\;180^{\circ }\right]}^{T}$ and was instructed the motion command, ${\left[V_{1}=0\;m/s,\;\phi {\;}_{=}\;0\;\mathrm{rad},\;\omega_{1}=0.154\;\mathrm{rad}/s\right]}^{T}$. According to the given motion command, the robot should be rotated around the center of block 1 (ICC lies in the center of block 1, $c_{1}$). The snapshots taken during the robot movements are given in Figure 8 along with the motion analysis results. According to the traced paths, the center of blocks almost rotated on circular paths centering $c_{1}$. However, a slight variation of $c_{1}$, which should have been a standstill, could be observed (4 cm). This variation of $c_{1}$ caused the minor deviations of the paths of $c_{2}$, $c_{3}$, and $c_{4}$ in the latter part of the movement. The hinge angles recorded from the encoders during the test case are plotted in Figure 9a. According to the plot, only slight deviations of hinge angles from the desired configuration could be observed (maximum deviation 3${}^{\circ }$), which suggests that the robot is capable of firmly maintaining the shape in this test case.

**Figure 10:**
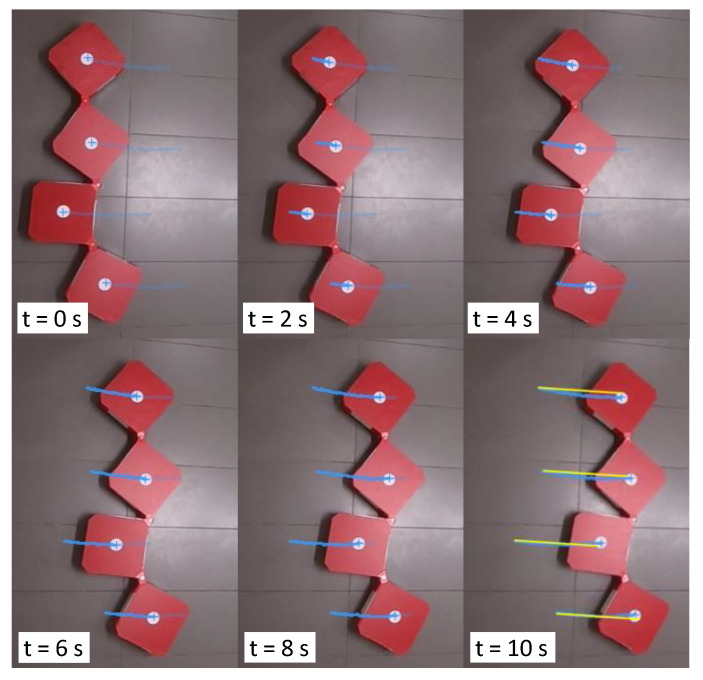
Snapshots taken, and the motion analysis during a locomotion with $V_{1}=0.045$ m/s, $\phi_{1}=-\frac{\pi }{4}$ rad, and $\omega_{1}=0$ rad/s. The traced paths are annotated in blue. The expected movement paths are given in yellow.

In the second sample test case, the robot was reconfigured to ${\left[80^{\circ }\;22^{\circ }\;64^{\circ }\right]}^{T}$ and was given the motion command ${\left[V_{1}=0.45\;m/s,\;\phi_{1}=-\frac{\pi }{4}\;\mathrm{rad},\;\omega_{1}=0\;\mathrm{rad}/s\right]}^{T}$. According to the command, the robot should be moved toward the right in a linear path without any rotations. The snapshots taken during the robot’s movement in this test case are given in Figure 10, along with the motion analysis results. According to the results, all the centers of the blocks moved in a linear path as expected. The variation of hinge angles during the movement is given in Figure 9b. The observed variation of hinge angles confirms the robot’s ability to maintain the shape firmly during the locomotion of this case (since deviations are minor). Similarly, 10 heterogeneous locomotion cases were considered. In all the test cases, the robot was moved as expected, and only minor deviations of the hinge angles could be observed (maximum deviation = 4${}^{\circ }$). Therefore, it can be concluded that the proposed robot model and controller are capable of navigating the robot per given instruction while firmly maintaining the shape configuration of the robot.

The deviations of the hinge angle could be further reduced by changing the error threshold defined for the selection between the reconfiguration handler and the locomotion handler. However, the reduction of the threshold causes unnecessary interruptions to the robot’s locomotion since the robot tries to attempt even a minor reconfiguration error while stopping the locomotion. The robot is not equipped with additional sensors to localize within the world frame. Particularly, the robot localization within the world frame is open-loop in this stage of the work. Hence, slight variations of the robot’s position and orientation (block 1 is the reference) with respect to the world frame could be experienced during the test cases. The robot should be equipped with additional sensors, an inertial measurement unit and a Lidar to have localization within the world frame during continuous operation. The establishment of localization within the world frame would allow closed-loop operation and correction of the localization errors. These developments are the immediate future work.

The controller of the robot should take necessary actions to maintain the hinge angles corresponding to a given shape configuration while locomoting the robot as required for the coverage strategy. If no control actions were made, the hinge angles would deviate from the required values since the hinges allow free movements (passive hinges and no locking mechanism). The prototype of hTetro used for this work has been designed to have an omni drive mechanism in each block for avoiding the singularities of the robot model when realizing this goal. Moreover, the use of omni drives simplifies the controlling. In contrast, the implementation cost of the robot would be high compared to the previous version of hTetro, which considered only seven discrete shapes for the reconfiguration (e.g., [25,26]). Nevertheless, the simulation outcomes of the work [28,29,30] have proven that the ability of hTetro to reconfigure and locomote with any shape can significantly improve the area coverage performance. The main intention of deploying the reconfigurable robots for floor cleaning is to realize complete area coverage performance. Thus, it would be worthy to tradeoff implementation cost against a significant performance improvement.

### 3.4. Discussion

In the present controller design, the reconfiguration and navigation are not simultaneously performed. If the desired morphology is lost during locomotion, the robot has to stop and reconfigure to the desired shape again before continuing the locomotion. This behavior would cause interruptions for locomotion when the robot locomotes on long courses. This decoupling of reconfiguration and locomotion is the main limitation of the proposed controller design. In the present stage of the work, it was assumed that the robot would have to locomote only shorter distances with shapes other than the primary four shapes. For example, the high-level autonomy strategies proposed in [28,29,30] expect the robot to travel 25 cm after reconfiguring into a shape to cover an obstacle. Therefore, the interruptions during the locomotion would not cause much overhead for the robot. Furthermore, this is the first work of a line of research that considers the development of low-level controlling required for reconfiguring into many shapes and locomoting while maintaining the shapes. Therefore, it is justifiable to design a suboptimal controller at the present stage of the research. A more robust and optimal controller that considers simultaneous reconfiguration and locomotion is expected to be designed in the future to resolve this limitation.

The robot reconfiguration and locomotion are performed considering block 1 as the reference, where the position and the heading of block 1 are considered as the robot’s position and orientation, respectively. During reconfiguration, the reconfiguration handler is capable of maintaining the required hinge angles with an adequate level of accuracy. The reconfiguration is performed with respect to block 1, and the position and orientation of block 1 should be accurate for the precise positioning of the robot in the world frame. However, a slight deviation of the position and the orientation of block 1 could be observed due to the forces acting on it due to the friction of hinges and other mechanical constraints. The angular velocity of reconfiguration should be feasible in satisfying the linear and angular velocity requirements of all the blocks. For example, when the robot is reconfigured around hinge 1, block 4 should have the highest linear velocity required to maintain the same angular velocity as blocks 2 and 3 around hinge 1 since the radius is highest. The maximum angular rotation of hinges during the reconfiguration is configured to 0.09 rad/s considering this requirement. With this configuration, the observed deviations of block 1 were less than 3 and 2 cm along with the robot’s Y-axis and X-axis, respectively, in the test cases. These observations suggest that the deviation of the robot’s position with respect to the world frame is slight. Furthermore, theses deviations can be corrected by a high-level autonomy layer that localizes the robot on the world frame. However, the robot’s localization in the world frame is not discussed within the scope of the work proposed in this paper. In other words, the robot’s position and orientation with respect to the world frame are not considered in this work. Implementation of a localization method in the autonomy layer to localize and correct the position and orientation with respect to the world frame is one of the essential future works.

The proposed robot design has four blocks that have individual locomotion modules. On the other hand, there can be four individual robots similar to a block of the proposed robot design as a swarm of robots. In swarm robots, each robot should be equipped with sensors for perceiving the environment and localization, such as Lidars and Inertial Measurement Units (IMUs). Furthermore, each robot should have controller hardware, such as microcontrollers and single-board computers, for processing work. In contrast, all the blocks of an intra-reconfigurable robot can share hardware resources for processing and sensing. Swarm robots should have wireless communication between robots (through a central unit or not) for proper accomplishment of a task such as cleaning where the units of an intra-reconfigurable do not require such communication functionality. Thus, the implementation cost of an intra-reconfigurable robot is less than that of a swarm of robots. In addition to that, an intra-reconfigurable robot has more power and flexibility to execute a cleaning task compared to a set of individual robots. These are the major advantages of intra-reconfigurable robots with respect to comparable swarm robots.

## 4. Conclusions

Reconfigurable robots have been introduced to cater to the demand in area coverage applications. The existing models and controllers of reconfigurable robots consider only a small set of predefined shapes for the reconfiguration. However, the consideration of a few shapes for the reconfiguration limits the performance of robots. The proper locomotion of a reconfigurable robot while maintaining a given shape configuration beyond a small set of predefined shapes requires complex modeling and controlling requirements.

A novel robot model and controller to achieve the low-level functionalities required to reconfigure and locomote a reconfigurable robot while maintaining a given shape configuration other than a predefined shape was proposed in this paper. The proposed robot model and controller have been integrated into a prototype of a reconfigurable robot. The experimental results validate that the proposed model and controller are capable of reconfiguring into any given shape configuration and subsequently locomoting per instructions while maintaining the shape configuration.

Moreover, the low-level functionalities required for achieving performance enhancement in high-level decision-making methods, which consider reconfigurations beyond a set of shapes to improve productivity, have been designed and developed in the scope of this paper. As future work, the high-level autonomy layers proposed in existing work limited to simulations are expected to be integrated with the robot model and controller proposed in this paper to achieve high-level autonomy abilities required for reconfiguration and locomotion, such as reconfiguring per context.

## Supplementary Materials

The following are available online at https://www.mdpi.com/article/10.3390/s21165362/s1.
